# Environmental hazard ingredients in women’s hair growth cosmeceuticals: a comprehensive analysis

**DOI:** 10.1097/JW9.0000000000000230

**Published:** 2025-10-03

**Authors:** Kritin K. Verma, Michelle B. Tarbox, Daniel P. Friedmann, Paige W. Wolstencroft, Misha Rosenbach

**Affiliations:** a Texas Tech University Health Sciences School of Medicine, Lubbock, Texas; b Department of Dermatology, Texas Tech University Health Sciences Center, Lubbock, Texas; c Westlake Dermatology and Cosmetic Surgery, Westlake Dermatology Clinical Research Center, Austin, Texas; d Department of Dermatology, Stanford University School of Medicine, Stanford, California; e Department of Dermatology, University of Pennsylvania, Philadelphia, Pennsylvania

**Keywords:** eco-friendly hair treatments, fragrance, hair growth cosmeceuticals, phenoxyethanol

## Abstract

**Background:**

Hair growth cosmeceuticals (HGCs) for women have become increasingly popular.

**Objective:**

We sought to analyze the ingredients of HGCs and their potential environmental hazards.

**Methods:**

A cross-sectional study was conducted using a Google Boolean search to identify the top 10 nonsponsored articles on HGCs. After applying exclusion criteria, 58 unique HGCs marketed as either unisex or female products were analyzed. Ingredients were documented from product websites, and HGCs were categorized based on specific ingredients with potential environmental concerns.

**Results:**

The study found that phenoxyethanol (32.8%) and synthetic fragrances (25.9%) were the most common ingredients with potential environmental implications, with trisodium Ethylenediaminetetraacetic acid (EDTA), trisodium ethylenediamine disuccinate, isobutane, butane, n-butane, ethylhexyl methoxycinnamate, and benzophenone-4 also found. Ingredients such as sodium lauryl sulfate, parabens, and microbeads were, however, not found in any of the products evaluated.

**Limitations:**

The study was limited by potential reporting and sampling bias.

**Conclusion:**

Significant environmental hazards, particularly to aquatic habitats, have been associated with specific ingredients commonly found in HGCs.

**What is known about this subject in regard to women and their families?**

- Hair growth cosmeceuticals (HGCs) are increasingly popular among women seeking to address hair thinning and loss.
- These products aim to stimulate hair follicles, increase hair density, and enhance scalp health.
- Women and their families may be unaware of the potential environmental impacts of the ingredients in these products.

**What is new from this article as messages for women and their families?**

- Many HGCs contain ingredients that may pose significant risks to aquatic habitats and air quality.
- Common ingredients of concern include phenoxyethanol (32.8% of products) and synthetic fragrances (25.9% of products).
- This information can help women and their families make more informed decisions about the hair care products they use, considering both personal and environmental health.
- The study highlights the need for more eco-friendly alternatives in hair care products, which may influence consumer choices and industry practices.

## Introduction

Cosmeceuticals for hair thinning and loss are becoming increasingly popular, intending to stimulate hair follicles, increase hair density, and enhance scalp health.^1,2^ Chemical ingredients in these products, however, may have a negative environmental impact, particularly on aquatic habitats from improper disposal.^3,4^ Given that no prior study has investigated this association, we sought to analyze the ingredients of common hair growth cosmeceuticals (HGCs) marketed as either unisex or female products and their potential environmental hazards.

## Methods

A Google Boolean search was performed in January 2024 using the following terms: list AND hair AND loss AND growth AND (serums OR solution). The top 10 nonsponsored results (online articles and magazines) were selected for this cross-sectional study from the 255,000,000 total results generated.^5–14^ Exclusion criteria included products marketed as shampoo and/or conditioners, meant solely for male use, or designed specifically for brows. After exclusion, 58 unique products were included. A literature review was performed to identify ingredients posing environmental concerns.

## Results

HGCs were categorized based on specific ingredients with potential environmental concerns (Table 1). Of the 58 HGCs analyzed, phenoxyethanol (*n* = 19, 32.8%) and fragrance/perfume (*n* = 15, 25.9%) were the most common. Ingredients such as sodium lauryl sulfate, parabens, triclosan, microbeads (polyethylene), and phthalates, among others, were not found in any of the products analyzed (Table 1). Thirty-two HGCs (55.2%) analyzed did not have any ingredients of environmental concern.

**Table 1:** Hair growth solutions and serums of environmental concern

Ingredient name	Potential environmental concern	Product containing analyzed environmental ingredient name
Phenoxyethanol (*n* = 19, 32.8%)	Toxic to aquatic life	Actsyl Hair Growth Serum
		Act + Acre Cold Processed Stem Cell Serum
		Alfaparf Semi Di Lino Scalp Renew Energizing Lotion
		Aveda Botanical Repair Strengthening Overnight Serum
		Better Not Younger Superpower+ Advanced Hair Densifying Scalp Serum
		BOLDIFY Hair Growth/Thickening Serum
		BosleyMD Follicle Energizer
		Curlsmith Scalp Stimulating Booster
		Davines Energizing Thickening Tonic
		dpHUE ACV Daily Scalp Serum
		Fable & Mane HoliRoots Hair Oil
		Monpure Follicle Boost Hair Density Serum
		OUAI Scalp Serum
		Pronexa Topical Hair Loss Serum
		Rene Furterer Triphasic Reactional Concentrated Serum
		Revela Hair Revival Serum
		Shapiro MD Hair Serum
		The INKEY List Caffeine Stimulating Scalp Treatment
		The Ordinary Multi-Peptide Serum for Hair Density
Fragrance/perfume (*n* = 15, 25.9%)	Potential for bioaccumulation and aquatic toxicity	Actsyl Hair Growth Serum
		Alfaparf Semi Di Lino Scalp Renew Energizing Lotion
		Aveda Botanical Repair Strengthening Overnight Serum
		BosleyMD Follicle Energizer
		Curlsmith Scalp Stimulating Booster
		Davines Energizing Thickening Tonic
		Fable & Mane HoliRoots Hair Oil
		Groh Stimulating Scalp Serum
		Kérastase Genesis Serum Anti-Chute Fortifiant
		KLORANE Strengthening Serum with Quinine and Organic Edelweiss for Thinning Hair
		Ouai Scalp Serum
		Pronexa Topical Hair Loss Serum
		Shapiro MD Hair Serum
		Stripes Ectoine Calming & Thickening Scalp Serum
		Weleda Revitalizing Hair Tonic
Trisodium EDTA, trisodium ethylenediamine disuccinate (*n* = 3, 5.2%)	Persistent and toxic to aquatic organisms	BosleyMD Follicle Energizer
		The INKEY List Caffeine Stimulating Scalp Treatment
		The Ordinary Multi-Peptide Serum for Hair Density
Isobutane, butane, n-butane (*n* = 2, 3.4%)	Volatile organic compounds contribute to air pollution	Virtue Hair Growth Treatment 1 Month Kit
		Women’s Rogaine 5% Minoxidil Foam or 2% Minoxidil Topical Solution
Ethylhexyl methoxycinnamate, benzophenone-4 (*n* = 2, 3.4%)	Potential endocrine disruptors, harmful to aquatic life and coral reefs	Alfaparf Semi Di Lino Scalp Renew Energizing Lotion
		Shapiro MD Hair Serum
Cocamide monoethanolamine (*n* = 1, 1.7%)	Potentially harmful to aquatic life	Shapiro MD Hair Serum
Sodium lauryl sulfate (SLS) (*n* = 0, 0%)	Toxic to aquatic organisms, nonbiodegradable	None
Parabens (eg, methylparaben, propylparaben) (*n* = 0, 0%)	Endocrine-disrupting effects, bioaccumulation	None
Triclosan (*n* = 0, 0%)	Persistent, bioaccumulative, and toxic to aquatic life	None
Microbeads (polyethylene) (*n* = 0, 0%)	Nonbiodegradable, can accumulate in the environment	None
Octocrylene, oxybenzone, homosalate, avobenzone, octinoxate (*n* = 0, 0%)	Potential endocrine disruptors, harmful to aquatic life and coral reefs	None
Methylisothiazolinone, methylchloroisothiazolinone (*n* = 0, 0%)	Toxic to aquatic life	None
Siloxanes (eg, cyclopentasiloxane, cyclotetrasiloxane) (n = 0, 0%)	Persistent, bioaccumulative, and toxic to aquatic life	None
Nonylphenol ethoxylates (*n* = 0, 0%)	Persistent, bioaccumulative, and toxic to aquatic life	None
Phthalates (eg, diethyl phthalate) (*n* = 0, 0%)	Endocrine disruptors, persistent in the environment	None
No identified environmental hazard of interest (*n* = 32, 55.2%)	None	Agent Nateur Holi(locks) Strengthening Hair Growth Serum
		Beneath Your Mask Nourish Skin & Hair Serum
		Better Roots RootBoost Serum
		Briogeo B. Well Organic + Cold-Pressed 100% Castor Oil
		Dr. Barbara Sturm Anti-Hair Fall Scalp Serum
		Dr. Berg’s (All In One) Hair Growth Serum
		Hair Thickness Maximizer Hair Serum
		hers Hair Regrowth Treatment
		Inala Power Potion
		Innersense Organic Beauty Natural Harmonic Treatment Oil
		Keeps Treatment Plan
		Keranique Hair Regrowth Treatment
		Kiehl’s Scalp and Hair Oil Treatment
		Liaison Hair Bond
		Lilivera 5% Minoxidil Spray
		Michele Green Hair Revitalizing Serum for Women
		Monat Hair Thinning Defense
		Nécessaire The Scalp Serum
		Nioxin Hair Regrowth Treatment for Women
		Nutrafol Growth Activator
		Nuele Nighttime Scalp Serum
		OPO Watercress Seed Oil
		Oway Densifying Remedy
		ProBliva Hair Growth Serum
		Pura D’or Hair Thinning Therapy Energizing Scalp Serum
		Rahua Founder’s Blend Scalp Treatment
		RANAVAT Fortifying Hair Serum
		Reverie CAKE Restorative Scalp Tonic
		TOMUM 5% Minoxidil Foam for Men and Women
		Vegamour GRO Hair Serum for Thinning Hair
		Vegnclever Hair Growth Serum
		Wild Growth Hair Oil

Ingredients are listed in descending order of frequency. Products containing each ingredient are listed alphabetically.

## Discussion

Our analysis determined that ingredients reported to have potential environmental hazards are commonly found in HGCs. Preservatives such as phenoxyethanol, methylisothiazolinone, and methylchloroisothiazolinone are commonly used in cosmeceuticals and have been purported to be toxic to fish and other aquatic organisms.^4,15^ Although the latter 2 were not found in HGCs in our analysis, 32.8% of products contained phenoxyethanol. Synthetic perfumes or fragrances were the second-most common ingredient (25.9%), with toxicity due to bioaccumulation in aquatic species reported to have a severe environmental impact.^16^ Trisodium EDTA and trisodium ethylenediamine disuccinate (5.2%) are chelating compounds that improve HGC stability and effectiveness. However, they are not readily biodegradable, remaining in the environment and accumulating in bodies of water, posing harm to aquatic organisms.^4,17^ Isobutane, butane, and n-butane in HGCs (3.4%) are propellants that act as volatile organic compounds (VOCs).^18,19^ VOCs are known to participate in photochemical processes that produce ground-level ozone, a major component of smog.^19^ A study found that VOCs from personal care products constitute a significant cause of urban air pollution, comparable to emissions from motor vehicles.^19^ Ultraviolet light filters like ethylhexyl methoxycinnamate and benzophenone-4 were found in 2 products (3.4%) and have been reported to induce coral bleaching and disturb marine organisms’ endocrine/reproductive processes.^20^ Cocamide monoethanolamine is a surfactant/emulsifier that was found in 1 product (1.7%) and may also pose a threat to aquatic life.^19^

Although some ingredients listed in Table 1 were not found in any HGC, other skincare or haircare products may contain these ingredients. Chemicals such as parabens, triclosan, and other ultraviolet filters have been found to bioaccumulate in aquatic wildlife, leading to endocrine disruption.^21,22^ Polyethylene microbeads with exfoliating properties are not biodegradable and can accumulate within the food chain of marine habitats.^22,23^

Our study is limited by potential reporting bias (errors or omissions) in the ingredient lists of the HGCs evaluated, as well as sampling bias, analyzing only the top 10 nonsponsored Google search results. While the provided table of products is by no means exhaustive, we hope it can serve as a primer to evaluate similar hair growth products across brands or manufacturers. This study may be useful to dermatologists in recommending more environmentally conscious options to patients attempting to use over-the-counter products to recover from hair loss, as well as for patients who are taking that initiative themselves. Another limitation of this table is that it does not include prices, which is a major factor for many patients trying to purchase safe and effective hair growth products. In addition, calculating estimates of potentially toxic product levels could be the goal of further studies or further product testing, as these estimates may rely on multiple factors outside the scope of our search results.

## Conclusion

Significant environmental hazards have been associated with specific ingredients commonly found in HGCs. Toxicity to air quality, coral reefs, and both the food chain and endocrine processes of aquatic organisms has all been implicated. Further research into the long-term environmental effects of these chemicals is needed, with the potential for more sustainable and eco-friendly alternatives to currently available HGCs.

## Conflicts of interest

None.

## Funding

None.

## Study approval

N/A

## Author contributions

KKV gathered the data and wrote the first draft. DPF analyzed the findings. All other authors made subsequent revisions.
